# Nurse leaders’ perspective on heat-related challenges and work-organizational interventions in inpatient care settings in Germany: A qualitative descriptive study

**DOI:** 10.1016/j.joclim.2026.100655

**Published:** 2026-04-23

**Authors:** Maria Zink, Franziska Jung, Steffi G. Riedel-Heller, Katharina M.A. Gabriel

**Affiliations:** a Federal Institute for Occupational Safety and Health (BAuA), Dresden, Germany; b University of Leipzig, Institute for Social Medicine, Occupational Health and Public Health, Leipzig, Germany; c Federal Institute for Occupational Safety and Health (BAuA), Berlin, Germany

**Keywords:** Disaster planning, Hospital, Long-term care, Health services administration, Nursing staff, Organizational learning, Patient care, Working conditions, Extreme heat event, Heat wave

## Abstract

**Background:**

In Germany, rising heat-related mortality and hospital admissions are posing significant challenges to healthcare systems. Nurses working in inpatient care facilities are particularly vulnerable because of heat stress.

**Objective:**

This qualitative descriptive study explores the impact of heat waves on nurses and their work environment in inpatient care facilities. It examines existing interventions to mitigate heat stress and identify areas for improvement. The study was conducted using semi-structured interviews with nurse leaders from inpatient care facilities, including hospitals and long-term care facilities in Saxony, Germany.

**Methods:**

Forty-eight nurse leaders from varied roles and facilities were interviewed (September 2022–June 2023). Qualitative analysis identified key themes around heat stress, impacts, and adaptations.

**Results:**

The majority of hospital nurse leaders and all long-term care facility nurse leaders reported significant challenges because of high outdoor temperatures. Heat stress negatively affects nurses’ physical health, cognitive function, and overall work performance while also impacting patient care and medication management. Facilities reported implementing 38 different interventions, with organizational and personal-level adaptations being more common than technical interventions. However, adaptation efforts remain fragmented.

**Conclusion:**

The study highlights the urgent need for structured heat adaptation in inpatient care, providing rationale for healthcare institutions to prioritize protections for nurses, patients, and residents amid rising temperatures. Future research should assess the effectiveness of current interventions, identify new solutions, and explore systemic barriers to successful implementation. To inform future policy and practice, the findings underscore the importance of formalizing heat action plans and integrating climate resilience into institutional strategies across the healthcare sector.

## 1 Introduction

Globally, 2024 was the warmest year on record [1]. In 2023, heat events were responsible for 47,000 deaths in Europe, with Germany being the country with the third highest number after Italy and Spain and a higher mortality rate (deaths per million) than France [2,3]. Germany is experiencing an increasing number of hot days (days when maximum air temperature exceeds 30 degrees Celsius) over the last three decades [4] and higher frequency of extreme heat events - periods characterized by sustained high temperatures and/or humidity levels that significantly exceed local historical averages [5,6].

As heat stress challenges the body’s thermoregulatory systems, heat poses a significant and increasing risk to human health. On the hottest five percent of days in Germany, hospital admissions increased by 25,000 compared to moderate-temperature days [7]. The exacerbation of chronic conditions (e.g., heart disease, respiratory issues, [8]) and mental health issues (e.g., anxiety, depression [9]) as well as the increasing incidences of heat-related illnesses (e.g., heat stroke, dehydration [10]) results in an overwhelming demand on emergency services [9,11] and necessitates an adaptation of patient care by nurses [12].

In addition to the extra workload, nurses working in inpatient facilities are subject to considerable occupational heat stress. A representative German health insurance survey based on self-reported and insurance data found that 23 percent of insured workers in all fields feel strongly burdened by outdoor heat during work hours, compared to nine percent during leisure time and 18 percent during daily tasks [13]. Heat stress during work is associated with concentration problems and a reduction of performance and productivity. Twenty percent of workers reported heat-related health issues, (e.g., exhaustion, sleeping disorders, extreme sweating, and circulatory problems), and 17 percent of respondents reported attending work despite feeling ill due to heat. Among surveyed professions, inpatient nurses, especially those working in geriatric care, were extremely vulnerable to heat stress, with 50 percent of the participating nurses experiencing high levels of heat stress [13]. Alongside construction workers, nurses experience the highest heat stress levels among all participants [13]. Occupational heat stress may vary between countries, as it is influenced by multiple factors such as the intensity of climate change, workforce characteristics, and the degree of societal adaptation.

In general, risk factors for occupational heat stress include working outdoors, being female or older, doing physical labor, working in protective equipment, or having a chronic illness [13,14]. However, indoor workers are also at risk when workplaces lack sufficient cooling or ventilation [14]. The nursing workforce in Germany is predominantly female and aging, with many individuals experiencing chronic health conditions [15] and performing physically demanding work with workload increasing during heat events. Nurses working in personal protective equipment during hot days are exposed to heat stress and found work exhaustive (96.5 percent), or had problems in breathing (93.0 percent) or in concentration (85.8 percent) [16]. Environmental risks also contribute, as many hospitals and long-term care facilities are housed in older buildings or have been renovated without sufficient adaptation to climate change [17]. Due to hygiene requirements, these workplaces often lack air conditioning [18]. In addition to these occupational, physiological and environmental risks, organizational risks also contribute, as nurses often face long shifts, staffing shortages, and limited breaks, which hinder adequate recovery [19,20]. Given that inpatient nurses already face significant work-related stress in Germany [21,22], heat further exacerbates their workload and poses a serious risk to their health.

To effectively address heat-related challenges and the increasing work stress among nurses, inpatient care facilities must implement adaptive interventions in response to the intensifying climate crisis. However, crisis management for heat events in Germany remains underdeveloped and insufficient [[23], [24], [25]], and studies on work-organizational interventions to reduce nurses’ occupational stress are scarce. Therefore, this study aimed to explore how nurse leaders of strategic (e.g., chief nursing officers), middle management (e.g., department head nurses) and operative (unit nurse managers) levels in inpatient care facilities in Saxony, Germany, perceive the challenges posed by extreme heat. We also aimed to identify the organizational interventions implemented to manage heat and reduce nurses’ occupational stress. To address the study’s purpose, we formulated two primary research questions to provide a comprehensive understanding of the current situation:

Q1: What challenges do high outdoor temperatures impose on inpatient nursing staff in acute and long-term care settings?

Q2: What interventions do inpatient care facilities implement to deal with those heat-related challenges?

To answer these questions, a qualitative descriptive approach using semi-structured interviews was chosen, as it was particularly suited for exploring emerging and under-researched phenomena [26]. Data were analyzed following descriptive qualitative content analysis.

In addition to the interview questions, a third question was addressed:

Q3: What other relevant aspects emerge in participants’ responses?

The third question ensures that, beyond the two predefined themes identified prior to the interviews, no significant issues raised by participants would be overlooked in data analysis. By incorporating this question, the study leverages the strengths of qualitative interviews, allowing for a more comprehensive and nuanced understanding of the topic.

## 2 Methods

### 2.1 Study design

The primary study objectives were to determine whether heat or heat events pose a challenge for inpatient care facilities and nursing staff, to identify the specific challenges encountered, and to assess the implementation of heat adaptation interventions using a descriptive qualitative design. Semi-structured interviews in German were conducted with nurse leaders from various management levels (e.g., strategic, middle, and operational) in inpatient care facilities, including hospitals and long-term care settings across Saxony (in eastern Germany). On average, Saxony experiences more hot days than regions in northern and parts of northwestern Germany, though areas such as Berlin, Brandenburg, and southwestern Germany record even higher numbers. Within Saxony, heat exposure varies considerably—from 13–16 hot days in the north to 7–12 in the south [27]. This regional variability, combined with Saxony’s high heat vulnerability as the state with the fifth oldest population [28], makes it a particularly relevant region for this study. The study was approved by the Ethics Committee of the German Federal Institute for Occupational Safety and Health (Research Project F2541; Approval Number 047/2021).

### 2.2 Recruitment of nurse leaders

Nurse leaders in inpatient facilities across different regions of Saxony (urban, non-metropolitan, rural), facility sizes, ownership types and management levels of nurse leaders were purposively sampled, with a targeted number of 48 participants (one nurse leader of strategic or middle management and two nurse leaders of operative management for each facility) from 16 facilities (eight per setting, balanced for size, region, and ownership), ensuring diverse perspectives. Two project members conducted recruitment using publicly available phone numbers and email addresses. They contacted 17 hospitals and 19 long-term care facilities, providing each with a study information letter. Following positive responses from 11 hospitals and 6 long-term care facilities (48 percent response rate), nurse leaders’ contact information was provided by the facilities. The nurse leaders were subsequently informed about the study and consent was obtained. Reasons for negative responses on facility-level were time restrictions, staff shortages, time required for nurse leaders to receive management’s approval for study participation, poor availability by phone or mail, or lack of approval of participation by hospital management. Three nurse leaders of one hospital dropped out because of illness of nurse leaders.

### 2.3 Data collection

The interview questions regarding heat-related interventions (Table 1) which guided research questions 1 and 2 were part of a larger qualitative study examining interventions implemented during the COVID-19 pandemic and were asked at the end of the interview [29]. Research question 3 guided the analysis and was therefore not asked directly in the interviews.

**Table 1:** Interview questions as documented by the interview guide to gather information on heat-related challenges and implemented interventions.

Interview structure	Standardized interviewers’ statements
Introduction	Germany experienced heatwaves and very high temperatures again this summer.
*Question 1*	*Do very high temperatures affect the work of nurses on your unit / in your residential unit / in your facility?*
Question 1a: If answer to Q1 was positive and participant did not yet explain	To what extent? How is the work of nurses affected?
*Question 2:* *If answer to Q1 was positive*	*What interventions are implemented by your facility / yourself / your team to adapt to heat-related challenges?*

The interview guide was piloted internally (one interview with member of the faculty but not part of the project team) and externally (two interviews with nurse leaders). Heat-related questions needed no revision. No interview needed repetition. All three interviewers (one male, two females) had prior experience conducting semi-structured interviews and were unknown to the participants. After interviewer training and mutual observations among the three interviewers during piloting and the first six interviews to standardize the guide’s use, 48 interviews (n = 30 nurse leaders of 10 hospitals, 18 nurse leaders of 6 long-term care facilities) were conducted between July 2022 and June 2023, and were recorded using a digital recorder and ranged 45–90 min. Interviews took place in a quiet and private room at the participants’ workplace, without others present during the interview. Facilities were distributed across regions, size and ownership types. Nurse leaders varied across management levels. Table 2 presents the sample composition in full detail.

**Table 2:** Characteristics of hospitals and participants.

Facility	Hospital					Long-term care				
Ownership	Public		Private	Charitable	Total	Public		Private	Charitable	Total
N	4		3	2	10	0		1	5	6

Level of care	level 1	level 2	level 3	specialist hospital	Total	/		/	/	/

N	4	3	1	2	10	/		/	/	/

Regional distribution	metropolitan		non-metropolitan		Total	metropolitan		non-metropolitan	rural	Total

N	6		4		10	2		2	2	6

Hospital size	min	max	M	SD	Total	min	max	M	SD	Total

Nurses	120	2000	666.7	607.3	8	48	142	86.4	37.9	5
Beds	165	1451	506.7	506.6	8	89	242	141.4	58.7	5

Participants										

Management level	strategic		middle management	operative	Total	strategic		middle management	operative	Total

n (%)	5 (16%)		8 (27%)	17 (57%)	30 (100%)	3 (17%)		6 (23%)	9 (50%)	18 (100%)

Education	Vocational degree		College/university degree	other	Total	Vocational degree		College/university degree	other	Total

n (%)	11 (48%)		8 (30%)	6 (22%)	27 (100%)	1 (6%)		6 (35%)	10 (59%)	17 (100%)

*Age group* (in years)	26-35	36-45	46-55	56-65	Total	26-35	36-45	46-55	56-65	Total

n (%)	4 (13%)	12 (40%)	8 (27%)	6 (20%)	30 (100%)	3 (17%)	5 (28%)	2 (11%)	8 (44%)	18 (100%)

Sex	female		male		Total	female		male		Total

n (%)	20 (67%)		10 (33%)		30 (100%)	12 (67%)		6 (33%)		18 (100%)

*Note.* n = number of participants; N = number of facilities; M = mean; SD = standard deviation.

### 2.4 Data analysis

The 48 audio files were transcribed verbatim and analyzed using descriptive qualitative content analysis in MAXQDA and MS Excel based on Kuckartz and Rädiker [30]. One researcher coded the interviews using deductive and inductive codes, and the final coding system as well as the results were discussed by the research team (Supplementary File 1). The first step involved identifying whether participants considered heat a challenge and why. In the second step, the type of challenge, promoting and hindering factors to address heat-related problems as well as implemented interventions, were coded. In this step, research question 3 was coded as well. Interventions were quantified regarding the number of participants stating the intervention and the answers were clustered by institutional affiliation to avoid overstating single interventions. Moreover, interventions were categorized according to the target group (nurses, patients, or both) and the (S)TOP-principle, a framework for occupational safety that prioritizes Substitution to Technical measures, and prefers them to Organizational measures, and Personal protective measures. Several quotes (content-equivalent German-to-English translation by the authors, verified through back-translation) were reported to illustrate these findings (Supplementary File 2); a detailed summary of data is provided (Supplementary File 3). Data saturation for the heat-related questions was reached after about 75% of the interviews were completed. However, data collection was continued because the heat-related items were embedded in a larger study design (see 2.3 and [29]).

### 2.5 Quality criteria of qualitative research

According to the Consolidated Criteria for Reporting Qualitative Studies (COREQ; [31]), qualitative studies must address several aspects to ensure rigor: a study design with a clear methodological framework and sampling strategy (see sections 2.1 – 2.3), a qualified research team, with researcher reflexivity acknowledged and supported through standardized use of the interview guide, ensured through training and mutual observation (2.3), systematic analysis conducted multiple researchers (2.4), and findings grounded in data and illustrated by participant quotes (2.4). The COREQ checklist is provided in Supplementary File 4.

## 3 Results

### 3.1 Q1: impact on nurses and their work environment

According to 90% of the nurse leaders working in hospitals (n_H_ = 27) and all the nurse leaders in long-term care facilities (n_L_=18), heat events present significant challenges in the nursing work environment (altogether, 93.75% of participants). High indoor temperatures in units or residential areas were reported (28–40°C in hospitals and 30–35°C in long-term care facilities). The concern mentioned was not restricted to nurses' personal issues but was assigned also to patients or residents, and to other heat effects such as patient load and management and medication management.

#### 3.1.1 Effects on nursing staff

Regarding the effects on nurses, two nurse leaders acknowledged the challenges of heat events but did not observe negative consequences for their staff. One noted that nurses enjoy working in warmer conditions, while the other emphasized that patients were more vulnerable to heat than nurses. However, 32 nurse leaders (n_H_ = 19, n_L_=13) reported negative consequences for nurses, including increased physical strain, excessive sweating to the point of requiring clothing changes, heightened stress and physical discomfort, reduced tolerance, decreased performance, concentration difficulties, and faster exhaustion or fatigue. Draft exposure also led to some nurses reporting illness. Ten nurse leaders (n_H_ = 8, n_L_=2) highlighted that working in personal protective equipment exacerbates these strains. One nurse leader highlighted synthetic work clothing as particularly uncomfortable during heat events.

“Because our workwear, which is partly synthetic, is not suitable for high temperatures,we will soon be making a change in this respect. Something thinner is coming. We have relaxed the dress code in the summer months when it really gets hot. This means that they can also wear something thinner and could – sort of – deviate from the work clothes, even if it's not hygienic, but they still have to wear an apron if it's necessary for personal hygiene. That was very well appreciated by staff.” – Residential area manager of a rural long-term care facility (LP06WB0136 Pos. 321) [translated by authors]

#### 3.1.2 Effects on patients or residents

Twelve nurse leaders (n_H_ = 10, n_L_=2) reported effects on individuals in care. Patients, particularly older adults and those with severe illnesses, tend to be more irritable, experiencing excessive sweating and an increased risk of dehydration. Similarly, two nurse leaders reported heat events affecting residents (e.g., risk of dehydration, cardiovascular issues, physically burdensome, especially when hot days occur early in the year) as well as their daily routine (e.g., fewer activities; less showering; especially irritating for people with dementia). One nurse leader explicitly described the situation as dangerous for patients.

"For the residents, especially the older ones [...] actually around May, June, when it starts, or even earlier, in April. And suddenly you have these hot days, 30 degrees in May. Cardiovascular problems and so on [...] So the problem is worse there." - Residential area manager of a rural long-term care facility(LP06WB0237, Pos. 268) [translated by authors]

#### 3.1.3 Other effects

Effects on patient load and management as well as on medication management were identified. One noted that during heat events, patient load increases, and hospitals struggle to transfer post-surgical patients to non-air-conditioned units, further complicating patient management. Three nurse leaders (n_H_ = 2, n_L_=1) mentioned having issues with storing medications in compliance with temperature regulations.

“Fortunately, we do have air conditioning, but of course, we still feel the effects—when it’s 40°C outside, it’s not exactly cool inside either. It doesn’t directly affect our work, but it does impact patient capacity. For example, we can admit fewer patients to the general unit because the third operating room doesn’t have air conditioning, and we cannot place a post-operative patient in a general unit at 40 degrees. So, while it doesn’t significantly affect our workflow, it does influence patient management." – Unit manager of an intensive care unit of a metropolitan hospital (KH09TL0207, Pos. 158) [translated by authors]

### 3.2 Q2: implemented interventions

Analyses of data from interview question 2 (interventions to tackle heat-related challenges) revealed a total of 38 distinct interventions across both settings (interventions_H_ = 23, i_L_=30, see Table 3). The identified interventions can be organized into two main categories: type of intervention (technical, organizational, personal) and target group of intervention (nursing staff, individual in care, or both).

**Table 3:** Technical, organizational and personal interventions for nurses, patients and residents and both reported by participants.

Interventions affecting…	n_H_	N_H_	n_L_	N_L_
nursing staff				

**O – organizational interventions**				
Adherence to scheduled breaks	1	1	0	0
Increased work breaks (preferably outdoors)	0	0	3	2
**P – personal interventions**				
On-body cooling (cooling packs in pockets, damp towels on the neck)	4	2	1	1
Increasing fluid intake	2	2	2	1
Lighter work clothing	1	1	5	4
Cold foot baths	1	1	1	1
Frequent changes of clothing	1	1	0	0
Increasing frequency of showers	1	1	0	0
Staff training on heat-related health and safety measures	1	1	0	0
Supervisory staff reminding team members to be mindful of heat exposure	1	1	0	0
Seeking cooling opportunities at home	0	0	1	1

individuals in care (patients, residents)				

**T – technical interventions**				
Provision of lightweight blankets for patients	1	1	0	0
**O – organizational interventions**				
Scheduled hydration rounds	2	2	4	4
Administration of intravenous fluids for surgical patients during waiting times	1	1	0	0
Adjustment of medications	0	0	2	2
Avoidance of outdoor activities	0	0	1	1
Minimizing exposure to drafts	0	0	1	1
Modification of meals	0	0	1	1
Monitoring the health status of residents	0	0	1	1
Reduction of activities for residents	0	0	1	1
Reduction of task expectations due to prioritization of hydration of residents	0	0	1	1
**P – personal interventions**				
Education of patients about heat-related precautions by staff	1	1	0	0

both nursing staff and individuals in care (patients, residents)				

**T – technical interventions**				
Daytime shading	8	5	6	4
Use of fans, mobile cooling devices (especially in staff rooms or critical areas)	6	6	6	5
Air conditioning (in staff rooms, on unit)	4	4	1	1
Implementation of thermal-insulated windows	1	1	1	1
Securing bed linens in windows for cooling purposes	1	1	0	0
**O – organizational interventions**				
Provision of beverages	11	10	14	6
Morning or nighttime ventilation and keeping windows closed during the day	4	3	7	4
Provision of ice cream (e.g., food truck, in long-term care also for residents)	4	3	5	3
Creating airflow through cross-ventilation	1	1	0	0
Outdoor stays in shaded areas	0	0	3	3
Development of formalized heat response protocols	0	0	2	1
Provision of fresh fruits (e.g., watermelon)	0	0	2	2
Adherence to heat warnings and dissemination of information	0	0	1	1
Increased staffing for outdoor supervision	0	0	1	1
Short breaks	0	0	1	1
Work schedule adjustments (e.g., reduced consecutive working days)	0	0	1	1

*Note.* n_H_ = participants working in hospitals; N_H_ = number of hospitals; n_L_ = participants working in long-term care; N_L_ = number of long-term care facilitites.

The sub-categories of the main category type of intervention follow the (S)TOP principle (Substitution-Technical-Organizational-Personal) for interventions in occupational safety and health. There were six technical solutions (k = 35 mentions), 22 organizational (k = 76), and 10 personal interventions (k = 23) mentioned by the participants to adapt work environment and work organization to the heat.

The second main category contains three sub-categories. These are interventions targeted at nurses, individuals in care (patients, residents), or both, as they alter the shared facility environment. Of the 38 interventions identified, 16 interventions targeted both nurses and individuals in care (i_H_ = 9, i_L_=14), 11 focused on nurses only (i_H_ = 9, i_L_=6), and 11 on individuals in care (i_H_ = 4, i_L_=8).

The interventions reflect a wide variety of approaches. Regarding the overall implementation of intervention across the entire study population, only 16 interventions were reported by participants of more than one facility. Twelve of the 38 interventions were mentioned by participants from both settings.

“Because this is an old part of the building, we added the wing 10 years ago and it gets very hot in summer. The rooms reach such high temperatures that we now have to consider what to do with the medication, […]. We have to rearrange things somehow, use other rooms. A heat concept is currently being developed, […] We already provide free drinks for our employees, so they don't have to buy them, and work clothes are being adjusted. We have more work clothes available, so we can use shorter work clothes and different materials. Air conditioning is unlikely to be installed, as the building is already finished and it would be difficult to retrofit.” - Nursing service manager of a rural long-term care facility (LP07PDL38, Pos. 111) [translated by authors]

### 3.3 Q3: other relevant aspects: challenges for heat adaptation

Further analysis of interview data revealed another two themes - the influence of infrastructural factors on heat stress and implemented interventions, as well as overall challenges to successful heat adaptation. Regarding the first theme, the four infrastructural conditions were windows in rooms, direct solar exposure, location within the building, and age of the building. For example, participants stated temperatures tend to be higher in units or residential areas on upper floors of the building, in old and unrenovated buildings, or in rooms with direct solar exposure or large glass windows. According to participants, working in windowless rooms—such as bathrooms for residents and nurses’ offices—was particularly hot and demanding (n_H_ = 8 of N = 6 hospitals; n_L_=4 of N = 2 long-term care facilities).

“[Heat waves are affecting us] definitely. We do have a central air conditioning system, but in the end, there are rooms facing south where the sunlight through the large windows is so intense that the rooms heat up significantly. In the summer, this really becomes a serious issue, no doubt about it. Some rooms are slightly cooler due to being on the shaded side, but here on this side […]. It basically feels like a greenhouse. Even the air conditioning system can’t keep up, which makes it quite stressful—for both the staff and the patients. Honestly, they didn’t really think the design of this new building through properly." – Unit manager of an intensive care unit of a non-metropolitan hospital (KH11TL0222, Pos. 298-302) [translated by authors]

In most cases, interventions such as air-conditioning or shading options were implemented to compensate for unfavorable infrastructural conditions. For example, nurses working in air-conditioned buildings, units, or rooms reported being less affected by high outdoor temperatures (n_H_ = 4 of N = 4 hospitals; n_L_=1 of N = 1 long-term care facilities). None of the long-term care facilities are fully air-conditioned, though one long-term care facility does have air-conditioned nurses’ offices. However, two nurse leaders of different hospitals reported that sometimes outside temperatures get so high that the air-conditioning becomes ineffective. One nurse leader specifically described the air-conditioning as dysfunctional. In many cases, fans or mobile cooling devices serve as substitutes for air-conditioning (n_H_ = 6 of N = 6 hospitals; n_L_ = 6 of N = 5 long-term care facilities).

“It's difficult because the building is only air-conditioned in some units. It is very sweaty. We also got drinks from the hospital, which was important. Each unit had a small fan or a slightly larger one. Of course, that's not enough.” – Unit manager from a non-metropolitan hospital (KH13TL0226, Pos. 184) [translated by authors]

Regarding shading options, participants described both outside and inside shading of windows as useful during heat events. Some facilities in both settings plan to improve shading soon. One nurse leader from a hospital without shading options noted that she uses bed linens to block sunlight at the windows.

“What doI wish for? That the hospital simply puts roller blinds on this side of the unit. What we do now is – don't laugh! – but we take a blanket and hang it between the windows. It all looks very much like wartime.” – Unit manager of a metropolitan hospital (KH17TL0148, Pos. 255) [translated by authors]

Where shading options and air-conditioning were not available options to adapt to the heat, other interventions were implemented. Therefore, reported interventions and challenges necessarily differ, making heat adaptation and corresponding interventions complex and individualized for each facility, unit, or residential area. For example, ventilation of rooms was cited as a challenge either because of the absence of windows in certain rooms, concerns about drafts affecting nurses and patients or residents, lack of insect screens on windows, and fire safety regulations requiring fire doors to remain closed.

“At night we opened the windows to cool down a bit, but then insects flew in en masse. That’s quite a stressful situation.” - Director of nursing of a non-metropolitan hospital (KH11PD20, Pos. 161) [translated by authors]

During the interviews, participants shared the complexity of heat adaptation, highlighting several challenges that hinder successful adaptation (second theme of Q3). Although not directly queried in the study, only two participants from one long-term care facility mentioned that there were plans to formalize heat adaptation interventions through a facility-wide heat action plan. The use of heat warnings was mentioned once. The primary challenge for successful heat adaptation cited was the significant financial costs of building renovations and the installation of outside shading or air conditioning, particularly in older buildings. In hospitals, hygiene regulations were often cited as a barrier to the installation of air-conditioning. Additionally, some participants noted that heat protection was not a primary focus of facility management. One nurse leader mentioned that the facility was aware of the issue but had not taken active measures to address it, while another stated that heat adaptation was just one of many problems with a lower priority. Furthermore, nurse leaders noted that heat adaptation measures such as morning ventilation (airing rooms by opening windows) and hydration rounds (scheduled checks to ensure patients’ fluid intake) are time-consuming and require additional working time.

“It has often been the case that periods of extreme heat have caused a great deal of stress, but before adequate measures could be taken, these periods were already over and we were back to dealing with [other] issues, only to be surprised by the heat again the following year. [...] I do think that this is a stressful factor, but I don't believe that it is currently the main stress factor in the nursing profession.” - Director of nursing of a metropolitan hospital (KH17PD47, Pos. 119) [translated by authors]

## 4 Discussion

This study assessed challenges for inpatient care facilities (e.g., long-term care facilities, hospitals) and their nursing staff caused by high outdoor temperatures. The nature of these challenges was described, and interventions currently implemented to mitigate these issues were examined.

### 4.1 Impact of heat events on nurses and their environment

With most of the nurse leaders responding in the affirmative to the first question, the results clearly demonstrate that high outdoor temperatures pose a significant challenge for nurses working in inpatient care facilities, negatively impacting health. The challenges were not only for nursing staff (e.g., physical strain, reduced performance and cognitive impairment, increased workload due to ventilation, monitoring of patients, hydration), but also for patients and residents (e.g., physical strain, higher stress levels), patient load (e.g., more hospital admissions), patient management (e.g., difficulties transferring patients), and medication management (e.g., inadequate cooling).

The identified challenges highlight the interconnectedness of various aspects of nurses' work systems. These results corroborate studies examining heat-related challenges faced by nurses in Germany (e.g., [13], [16]). Nurses often feel unprotected from heat at work, with hospital nurses reporting the lowest levels of protection compared to those in nursing homes or outpatient services [16]. Additionally, hospital nurses were also least likely to report the presence of heat warnings or formal heat action plans in their institutions [16]. These findings align with global evidence of substantial health burdens associated with the climate crisis, which are already impacting workers today [5,13,32] and emphasize the dual burden of heat exposure and pre-existing high work stress among inpatient nurses.

### 4.2 Occupational hazard and risk assessment

While this study did not include temperature measurements, self-reports of high indoor temperatures ranging 28–40°C are concerning. According to German occupational safety regulations and the “Technical Rules for Workplaces – Room Temperature” (ASR 3.5; [33]), temperatures above 26°C in indoor workplaces with proper shading systems necessitate health-protective interventions based on German work risk assessment [34]. Such interventions may include reducing internal thermal loads (e.g., operating electrical devices only when necessary), early morning ventilation, flexible scheduling to shift working hours, relaxing clothing regulations, or additional cooling breaks. If the indoor temperature exceeds 30°C, intervention is required, and if the temperature exceeds 35°C, working in the room becomes impossible, unless the work is specifically organized to meet the requirements of working in the heat [33,35].

Further research is necessary to quantify the extent of this occupational hazard for nurses. It is essential that those responsible for workplace safety be made aware of the risks posed by heat to their nursing staff and potential countermeasures. In this context, carrying out a work risk assessment [34], [36] can be valuable.

### 4.3 Implemented interventions and infrastructural factors

The technical rule in question does not specifically address inpatient care facilities, nor does it provide an exhaustive list of possible interventions. This is particularly evident when considering recommendations such as implementing flexible work hours or avoiding work in rooms where temperatures exceed 35°C, especially when patients or residents require immediate care. Therefore, the interventions identified in this study provide a more comprehensive overview of feasible interventions during heat events. In total, nurse leaders describe 38 different interventions to tackle heat-associated challenges (n_H_ = 23, n_L_ = 30). These can be categorized into technical, organizational, and personal-level interventions, targeting nursing staff, patients/residents, or both by altering the shared environment. There are some overlapping interventions identified for the management of COVID-19 and heat stress [37]. Fewer than half of the identified interventions are mentioned in multiple facilities. The findings reveal substantial variability across facilities, indicating a high degree of individual heat adaptation. This aligns with the observations on the influence of infrastructural factors. Infrastructural factors (e.g., solar exposure, location of working area) influence indoor temperature and, thereby, the necessity of interventions, underlining the complexity of the issue.

In line with European Occupational Health and Safety law (EU-Directive 89/391/EEC), technical interventions should be prioritized, with personal-level interventions implemented only as a last resort [38]. However, the majority of reported interventions in hospitals were at the personal level, whereas long-term care facilities prioritized organizational interventions. In hospitals, most interventions were those affecting nurses. In long-term care settings, the majority of interventions affects the work environment, e.g., nurses and patients/residents. Compared to hospitals, long-term care facilities reported twice as many interventions targeted at patients or residents. Although fewer in number, participants from long-term care settings reported about 1.5 times more interventions than hospital participants. These differences may stem from the existence of a model heat protection plan for long-term care facilities [39], which recommends various technical, organizational, and resident-specific interventions (e.g., shading, ventilation, medication management, and hydration). The evidence suggests that these recommendations have been acknowledged and have prompted initial adaptations in work organization and environment. However, recommendations to protect nurses' health are lacking in the model plan.

### 4.4 Indications for future heat adaptation

The findings on interventions and infrastructural factors contribute to the existing body of knowledge on heat adaptation and highlight key areas for improvement in inpatient care settings [[39], [40], [41], [42]]. The absence of heat action plans and the lack of mention of heat warnings align with the findings of Jegodka et al. [16]. Despite the challenges posed by heat to nurses, patients, and the provision of care, the limited adoption of formal strategies, such as heat action plans, and the variety of individual interventions indicate that adaptation measures remain fragmented and insufficient. The wide range of identified challenges and interventions suggests that approaching the issue requires diverse strategies, which may themselves pose a barrier to effective implementation. Additional barriers to effective adaptation include financial constraints, outdated infrastructure (e.g., old buildings, lack of air conditioning), hygiene and fire protection regulations, and practical challenges such as the absence of insect screens, all of which hinder effective cooling of indoor spaces. Furthermore, competing management priorities limit the ability to prioritize heat adaptation activities. Overcoming these barriers requires proactive, facility-wide strategies and prioritization of heat resilience in management frameworks to ensure that both nursing staff and patients are protected from heat-related challenges. The study supports calls from various stakeholders in Germany for heat protection to become a mandatory component of healthcare facilities’ responsibilities [43]. Strengthening adaptation interventions is crucial not only to mitigate occupational stress and for patient safety—as patients are often at high risk due to their health conditions— but also for safeguarding nurses’ health, an urgent concern given the ongoing staffing shortages in Germany’s healthcare sector [44].

### 4.5 Professional dedication and motivation of nursing staff to adapt to heat

Though nurses and nursing management face multiple obstacles in adapting to heat, participants still described a broad variety of interventions. Since many of the mentioned obstacles are beyond the control of nurses and their leaders, interventions focus on organizational and personal interventions. Those observations demonstrate, on one hand, the perceived and varying individual burden of heat and the extensive impact on nurses’ work systems, and on the other hand, their motivation and proactivity of their actions, as well as their creativity, flexibility, and professional dedication. The latter is particularly evident in interventions such as on-body cooling or securing bed linens in patient windows. Nurses often implemented these measures independently, without direct instructions or hospital regulations, although nurse leaders emphasized that some necessary interventions demand additional time from nurses. This increases workload and exacerbates stress, making heat adaptation a complex issue to address. Ongoing efforts to enhance and formalize heat adaptation should consider these factors. For example, formalized plans should consider recommendations that invite nurses to individually adapt to their work area. This enhances existing intrinsic motivation and participation while leaving room for flexible reactions as the participation of nursing staff is considered to be important for successful crisis management [29,45].

### 4.6 Limitations and strengths

Sample heterogeneity contributes significantly to the robustness of qualitative findings [46,47]. The study’s diverse sample and in-depth qualitative approach provide valuable insights into heat-related challenges and interventions across different inpatient care contexts. The development of adaptation to and preparedness for heat events in hospitals and long-term care facilities is seen as relevant to nurse leaders. At the time of data collection, a model heat adaptation plan existed for long-term care facilities [39], addressing aspects such as communication, heat warnings, interventions for nursing and social service, cooling, training, or food service. Similar information and recommendations are publicly available [41,48,49]. For hospitals, a model heat adaptation plan was developed by Aktionsbündnis Hitzeschutz Berlin [40] and reissued by the German Federal Ministry of Health [50] after this study’s data collection, which may have led to further developments in hospital heat adaptation since, potentially affecting the validity of the findings. However, there is no evidence that heat adaptation has been widely implemented in German hospitals [43,51,52]. Thus, the results remain timely. The focus on nursing staff, the variety of interventions identified, and the indicators for improving heat adaptation and overcoming barriers provide valuable insights that can help enhance existing heat adaptation plans and activities.

Study results are limited to insights of inpatient facilities in Saxony and may not reflect experiences or interventions elsewhere in Germany. Additionally, while interviews provide diverse qualitative data, they rely on self-reported perceptions and were asked at the end of the overall interview, which may introduce bias. Furthermore, some interventions might not be reported as they are understood as expected adaptation in everyday work. As occupational safety and health of nurses was the subordinate objective of this study, only nurse leaders were interviewed. These limitations need to be tackled by a nationwide evaluation that quantifies challenges and implements interventions with the direct involvement of nursing staff and experts of other disciplines to further and comprehensively improve facilities’ preparedness.

### 4.7 Practical implications

This study underscores the urgent need for targeted interventions to address heat-related occupational hazards, particularly in a workforce already burdened by staffing shortages and increasing patient demands. The study provides practical examples for inpatient facilities to improve their response to heat-related challenges and mitigate nurses' occupational hazards, and can extend existing model heat adaptation plans with relevant interventions for nurses’ working conditions and health.

The widespread acknowledgment of heat-related challenges and the limited adoption of formal heat action plans highlight the need for systematic institutional interventions and strategies to address heat-related risks, including facility-wide heat adaptation strategies, with a focus on nurses’ health, safety, and work environments. Structural barriers such as financing, infrastructure, and regulations should be addressed to ensure effective adaptation. Heat adaptation can only be implemented successfully when it is recognized as a shared responsibility among stakeholders within and outside healthcare facilities, including collaboration between sectors such as healthcare, construction, and climate science. Policymakers and healthcare administrators should integrate climate adaptation strategies into healthcare policies to support facilities, healthcare workers, patients, and residents. Nurses should be involved at every stage of this process, including identification, decision-making, development, and implementation of interventions.

### 4.8 Implication for future research

Future research should assess and quantify the ongoing development of heat adaptation strategies across inpatient facilities, ideally through nationwide evaluations to allow for broader generalizability. Further research should include the perspectives of practicing nurses and relevant stakeholders (e.g., hygiene, communication, nursing and medical management, technicians, logistics, therapists, physicians, and social workers) both within and outside the facilities. To further develop feasible interventions, research should also focus on evaluating the effectiveness of identified technical, organizational, and personal interventions in reducing nurses’ work stress in a randomized study or epidemiological study and exploring new solutions for heat adaptation.

At the time of the study, a model heat adaptation plan was only available for long-term care facilities, while one for hospitals was under development. So far, there is no knowledge on the implementation in practice, but findings of this study hint that the implementation rate is low. Recently, more institutions have committed themselves to improving heat adaptation in long-term care facilities (e.g., [41], [42], [53], [54]). As interventions in hospitals tend to be more personal than technical or organizational, the need for more research on organizational interventions in hospital settings is indicated.

## 5 Conclusion

High outside temperatures significantly impact inpatient nurses’ work environments. The findings highlight the urgent need to identify effective targeted interventions to proactively mitigate heat-related challenges and occupational hazards in a workforce already strained by staffing shortages and increasing patient demands during heat events.

The study reveals a wide array of interventions implemented across hospitals and long-term care facilities. However, most interventions were informal and varied significantly across institutions. Technical interventions remain limited because of financial and infrastructural constraints. Organizational interventions were more common, but their implementation was inconsistent.

A key concern is the lack of formalized heat action plans. Facility-wide heat adaptation policies that prioritize nurses’, patients’, and residents’ safety are needed. The study also highlights systemic barriers that hinder comprehensive adaptation efforts.

To tackle these factors and ensure sustainable and effective heat adaptation in view of an intensifying climate crisis, healthcare policymakers and administrators, as well as healthcare facilities, should integrate climate resilience into institutional strategies. Collaborative efforts between healthcare facilities and their employees and policy sectors are essential to addressing heat-related challenges. Ultimately, the perspective of practicing nurses must be integrated in research and in intervention implementation processes.

## Glossary

n: number of participants;
n_H__:_ number of participants working in hospitals;
n_L__:_ number of participants working in long-term care facilities;
N: number of facilities;
k: number of times a intervention was mentioned across all participants

## Research data for this article

Research data within data protection requirements is available upon request. Please contact the corresponding author of this article.

## Funding

This work was supported by the Federal Institute for Occupational Safety and Health, Germany (Project number F 2541 “Interventions focusing on work organization as a part of pandemic management in inpatient care”).

## CRediT authorship contribution statement

**Maria Zink:** Writing – review & editing, Writing – original draft, Visualization, Project administration, Methodology, Investigation, Formal analysis, Data curation, Conceptualization. **Franziska Jung:** Writing – review & editing, Supervision. **Steffi G. Riedel-Heller:** Writing – review & editing, Supervision. **Katharina M.A. Gabriel:** Writing – review & editing, Visualization, Supervision, Methodology, Formal analysis, Conceptualization.

## Declaration of competing interest

The authors declare that they have no known competing financial interests or personal relationships that could have appeared to influence the work reported in this paper.

## Appendix Supplementary materials

Supplementary files Image, application 1

File 1_ final codesystem Image, application 2

File 2_Original German Verbatim Quotes Image, application 3

File 3_full data_enl Image, application 4

File 4_COREQ Checklist Image, application 5

## Footnotes

Supplementary material associated with this article can be found, in the online version, at doi:10.1016/j.joclim.2026.100655.
