# Supplementary material for: Nurse leaders’ perspective on heat-related challenges and work-organizational interventions in inpatient care settings in Germany: A qualitative descriptive study
Source: J Clim Chang Health. 2026 Apr 23;28:100655. doi: 10.1016/j.joclim.2026.100655 (PMC13127614; doi:10.1016/j.joclim.2026.100655)
Supplement: Supplementary file 1 — Supplementary files [file mmc1.pdf]

Authors' contributions

Transparency Statement According to CRediT Roles

For transparency, authors must submit a file outlining individual contributions to the paper for all authors and named contributors using the relevant CRediT roles: Conceptualization; Data curation; Formal analysis; Funding acquisition; Investigation; Methodology; Project administration; Resources; Software; Supervision; Validation; Visualization; Roles/Writing - original draft; Writing - review & editing. https://www.elsevier.com/researcher/author/policies-and-guidelines/credit-author-statement

Author	Contribution	Notes
Maria Zink	Conceptualization, Visualization, Investigation, Formal analysis, Methodology, Data curation, project administration<br><br>Roles/Writing: Original Draft; review & editing	--
Prof.in Steffi G. Riedel-Heller	Conceptualization, Supervision<br><br>Roles/Writing: Review & Editing	--
Dr. Franziska Jung	Conceptualization, Supervision	--

	Roles/Writing: Review & Editing	
Dr. Katharina Gabriel	Conceptualization, Supervision, Methodology, Formal Analysis, Visualization<br><br>Roles/Writing: Review & Editing	--
