# Supplementary material for: Nurse leaders’ perspective on heat-related challenges and work-organizational interventions in inpatient care settings in Germany: A qualitative descriptive study
Source: J Clim Chang Health. 2026 Apr 23;28:100655. doi: 10.1016/j.joclim.2026.100655 (PMC13127614; doi:10.1016/j.joclim.2026.100655)
Supplement: Supplementary file 2 — File 1_ final codesystem [file mmc2.pdf]

Supplementary File 1

Codesystem with Number of Codes

Main Category	Subcategory	Number of Codes Study Sample	Number of Codes Hospital	Number of Codes Long-Term Care
Challenges		115	74	41
	yes	44	26	18
	no	4	4	0
	nursing staff	34	21	13
	patients	13	10	3
	management	4	2	2
	degree	9	7	2
	other	7	4	3
Infrastructural factors		26	13	13
	floor building	10	6	4
	windowsless	2	0	2
	age of building	5	3	2
	other	9	4	5
Interventions		134	58	76
Technical		35	21	14
	Provision of lightweight blankets for patients	1	1	0
	Installation or use of air conditioning in staff rooms	5	4	1
	Use of fans, mobile cooling units (especially in staff rooms or critical areas)	12	6	6
	Daytime shading (ongoing improvements to shading conditions)	14	8	6
	Implementation of thermal-insulated windows	2	1	1
	Securing bed linens in windows for cooling purposes	1	1	0
Organizational		76	24	52
	Adherence to heat warnings and dissemination of relevant information	1	0	1
	Adherence to scheduled breaks	1	1	

	Adjustment of medications	2	0	2
	Administration of intravenous fluids for surgical patients during waiting times	1	1	0
	Availability of ice (e.g., through a mobile cocktail cart)	9	4	5
	Avoidance of outdoor activities	1	0	1
	Creating airflow through cross-ventilation	1	1	0
	Development of formalized heat response protocols	2	0	2
	Increased breaks (preferably outdoors)	3	0	3
	Increased staffing for outdoor supervision	1	0	1
	Minimizing exposure to drafts	1	0	1
	Modification of meals	1	0	1
	Monitoring the health status of individuals	1	0	1
	Morning ventilation (requires additional time) and keeping windows closed during the day	3	1	2
	Nighttime ventilation and keeping windows closed during the day	8	3	5
	Outdoor stays in shaded areas	3	0	3
	Provision of beverages	25	11	14
	Provision of fresh fruit (e.g., watermelon)	2	0	2
	Reduction of activities for residents	1	0	1
	Reduction of task expectations; prioritization of hydration in caregiving	1	0	1
	Scheduled hydration rounds (requires additional time)	6	2	4
	Short breaks	1	0	1
	Work schedule adjustments (e.g., reduced consecutive working days)	1	0	1
Personal		23	13	10
	Cold foot baths	2	1	1
	Increased fluid intake (staff)	4	2	2
	Increased frequency of showers	1	1	0
	Frequent changes of clothing	1	1	0
	Lighter work clothing (adhering to hygiene requirements where necessary)	6	1	5

	Use of cooling packs in lab coat pockets	1	1	0
	Use of damp towels on the neck for cooling	4	3	1
	Staff training on heat-related health and safety measures	1	1	
	Supervisory staff reminding team members to be mindful of heat exposure	1	1	0
	Education of patients about heat-related precautions by caregiving staff	1	1	0
	Seeking cooling opportunities at home	1		1
Other: Interventions		2	1	1
Other: mentioning of topics	Ventilation options	19	11	8
	shading options	17	11	6
	air-conditioning	40	23	17
	mobile fans	11	6	5
