# Supplementary material for: Nurse leaders’ perspective on heat-related challenges and work-organizational interventions in inpatient care settings in Germany: A qualitative descriptive study
Source: J Clim Chang Health. 2026 Apr 23;28:100655. doi: 10.1016/j.joclim.2026.100655 (PMC13127614; doi:10.1016/j.joclim.2026.100655)
Supplement: Supplementary file 3 — File 2_Original German Verbatim Quotes [file mmc3.pdf]

Supplementary File 2

Original German Verbatim Quotes

Original	Translation by authors
Also wir haben zum Glück eine Klimaanlage, aber ja natürlich merken wir das auch, wenn es draußen 40 Grad sind, ist es auch hier drin nicht mehr kalt. Unsere Arbeit beeinflusst in dem Sinne nicht, aber das Patientenaufkommen beeinflusst es. Also wir können weniger auf Normalstation legen, weil die im dritten OG ohne Klimaanlage sind und wir einen frisch operierten nicht bei 40 Grad auf die Normalstation legen können. Also das beeinflusst es schon und so einen Arbeitsablauf an sich weniger. (KH09TL0207 - intensiv, Pos. 158)	*"Fortunately, we do have air conditioning, but of course, we still feel the effects—when it's 40 degrees Celsius outside, it's not exactly cool inside either. It doesn't directly affect our work, but it does impact patient capacity. For example, we can transfer fewer patients to the general unit because the third floor room doesn't have air conditioning, and we can't place a post-operative patient in a general unit at 40 degrees. So, while it doesn't significantly affect our workflow, it does influence patient management." – unit manager of an intensive care unit of a metropolitan hospital (KH09TL0207 - intensiv, Pos. 158)*
Weil nämlich unsere Berufsbekleidung, die teilweise synthetisch ist, für hohe Temperaturen nicht geeignet ist. Wir haben demnächst dort noch eine Änderung dahingehend. Es soll was Dünneres kommen. Wir haben in den Sommermonaten, wenn das richtig reindonnert, die Kleiderordnung gelockert. Das heißt, die können auch was Dünnes anziehen und könnten sozusagen, da weichen wir ab von der Berufsbekleidung, auch wenn das von der Hygiene her nicht so ist, dann müssen sie halt doch eine Schürze anziehen, wenn es bei der Körperpflege notwendig ist. Das ist sehr gut angekommen. (LP06WB0136, Pos. 321)	*"Because our workwear, which is partly synthetic, is not suitable for high temperatures. We will soon be making a change in this respect. Something thinner is coming. We have relaxed the dress code in the summer months when it really gets hot. This means that they can also wear something thinner and could – sort of - deviate from the work clothes, even if it's not hygienic, but they still have to wear an apron if it's necessary for personal hygiene. That was very well appreciated by staff."* – residential area manager of a rural long-term care facility (LP06WB0136, Pos. 321).
„Definitiv [beeinflussen uns Hitzewellen]. Also wir haben zwar eine Klimaanlage, eine zentrale. Aber letztendlich gibt es bei uns Räume zur Südseite hin, wo die Sonneneinstrahlung durch die großen Fenster so extrem ist, dass sich die Räume dermaßen	*"[Heat waves are affecting us] definitely. We do have a central air conditioning system, but in the end, there are rooms facing south where the sunlight through the large windows is so intense that the rooms heat up significantly. In the summer, this really*

aufheizen, das ist wirklich im Sommer ein richtiges Problem, muss man schon so sagen, ne? Es gibt Räume, die sind etwas KÜHLER, durch die Schattenseite, aber hier gerade auf der Seite […]. Das wirkt wie so ein Gewächshaus letztendlich, ne? Da kommt auch die Klimaanlage nicht an, das ist dann schon belastend. Also sowohl für das Personal als auch für die Patienten, ne? Also da hat man einfach durch/ bei der Konstruktion dieses Neubaus nicht richtig nachgedacht, das muss man sagen, ne? (KH11TL0222 - intensiv, Pos. 298-302)	*becomes a serious issue, no doubt about it. Some rooms are slightly cooler due to being on the shaded side, but here on this side […]. It basically feels like a greenhouse. Even the air conditioning system can't keep up, which makes it quite stressful—for both the staff and the patients. Honestly, they didn't really think the design of this new building through properly."* – (KH11TL0222 - intensiv, Pos. 298-302)
Es ist schwierig, weil das Haus ist nur auf manchen Stationen klimatisiert. Es war sehr schweißtreibend. Wir haben dann die Getränke auch bekommen vom Haus, das war schon wichtig. Jede Station hat einen kleinen Ventilator gehabt oder einen etwas Größeren. Das ist natürlich nicht ausreichend. (KH13TL0226 - 2 M COVID, Pos. 184)	*"It's difficult because the building is only air-conditioned on some units. It is very sweaty. We also got drinks from the hospital, which was important. Each unit had a small fan or a slightly larger one. Of course, that's not enough."* – unit manager from a non-metropolitan hospital (KH13TL0226 2M COVID, Pos. 184)
Ja, weil das ist ein alter Gebäudeteil hier, aber wir haben vor 10 Jahren den Trakt angebaut und der ist im Sommer sehr heiß. Die Linkszimmer haben solche Temperaturen, dass wir jetzt zum Beispiel jetzt überlegen müssen, was wir mit den Medikamenten machen. Wir müssen irgendwie umräumen, andere Räume nutzen. (LP07PDL38, Pos. 112)	*"Yes, because this is an older part of the building, but we added a new wing 10 years ago, and it gets very hot in the summer. The rooms on the left side reach such high temperatures that we now have to consider what to do with the medications. We need to rearrange things somehow and use other rooms." – nurse leader of middle management of rural long-term care facility (LP07PDL38, Pos. 112)*
Wünschten? Dass einfach Rollos an diese Feldseite kommt. Was wir jetzt machen ist / nicht lachen, aber wir nehmen eine Bettdecke und hängen die dazwischen, zwischen das Fenster. Sieht alles sehr nach Kriegszeiten so ungefähr aus. (KH17TL0148 Covid-19 / Innere, Pos. 255)	*"What I wish for? That the hospital simply puts roller blinds on this side of the unit. What we do now is – don't laugh! – but we take a blanket and hang it between the windows. It all looks very much like wartime."* – (KH17TL0148, COVID-19 / internist., Pos. 255)
Nachts hat man ja die Fenster aufgemacht, damit man mal ein bisschen kühlen konnte, dann sind aber die Insekten in Massen reingeflogen, das ist schon eine belastende Situation. (KH11PD20, Pos. 161)	*"At night we opened the windows to cool down a bit, but then insects flew in en masse. That's quite a stressful situation."* (KH11PD20, Pos. 161)
[…] weil das ist ein alter Gebäudeteil hier, aber wir haben vor 10 Jahren den Trakt angebaut und der ist	*"Because this is an old part of the building, but we added the wing 10 years ago and it gets very hot in*

im Sommer sehr heiß. Die Zimmer haben solche Temperaturen, dass wir […] jetzt überlegen müssen, was wir mit den Medikamenten machen. Wir müssen irgendwie umräumen, andere Räume nutzen. Das wird auch gerade ein Hitzekonzept entwickelt, […]. Wir haben sowieso für unsere Mitarbeiter freie Getränke. Also das müssen die nicht kaufen und Dienstkleidung wird angepasst. Wir haben mehr gestellte Dienstkleidung, dass wir kürzere Dienstkleidung und anderes Material verwenden. Klimaanlage wird wohl nicht werden, da das Haus fertig gebaut ist und nachzurüsten wäre schwierig. (LP07PDL38, Pos. 111)	*summer. The rooms reach such high temperatures that we now have to consider what to do with the medication, […]. We have to rearrange things somehow, use other rooms. A heat concept is currently being developed, […] We already provide free drinks for our employees. So they don't have to buy them, and work clothes are being adjusted. We have more work clothes available, so we can use shorter work clothes and different materials. Air conditioning is unlikely to be installed, as the building is already finished and it would be difficult to retrofit." (LP07PDL38, Pos. 111)*
aber bisher war es oft so, dass die Zeit der großen Hitze zwar eine große Belastung mitgebracht hat, aber bevor dann reagiert wurde adäquat darauf, war diese Zeit schon wieder vorbei und wir haben uns schon wieder mit [anderen] Themen beschäftigt, um im nächsten Jahr […] aufs Neue, von der Hitze überrascht zu sein, aber […] Ich denke schon, dass das ein belastender Faktor ist, der jetzt aber aktuell glaube ich nicht den hauptbelastenden Faktor der Profession Pflege ausmacht. (KH17PD47, Pos. 119)	*"It has often been the case that periods of extreme heat have caused a great deal of stress, but before adequate measures could be taken, these periods were already over and we were back to dealing with [other] issues, only to be surprised by the heat again the following year, but [...] I do think that this is a stressful factor, but I don't believe that it is currently the main stress factor in the nursing profession."* (KH17PD47, Pos. 119)
Bei den Bewohnern, und auch gerade bei den älteren Bewohnern […] dort eigentlich die Zeiten so Mai, Juni, wenn es losgeht oder sogar noch früher, April. Und du hast auf einmal so heiße Tage, 30 Grad im Mai. Herz-Kreislauf und so weiter […] Also dort ist die Problematik schlimmer. (LP06WB0237, Pos. 268)	*"For the residents, especially the older ones [...] actually around May, June, when it starts, or even earlier, in April. And suddenly you have these hot days, 30 degrees in May. Cardiovascular problems and so on [...] So the problem is worse there."* (LP06WB0237, Pos. 268)
